# Peroxidase is a novel potential marker in glioblastoma through bioinformatics method and experimental validation

**DOI:** 10.3389/fgene.2022.990344

**Published:** 2022-08-31

**Authors:** Weiwei Shi, Wenjie Ding, Zixuan Zhao, Rui Wang, Fengxu Wang, Yanfen Tang, Jinfeng Zhu, Chengcheng Su, Xinyuan Zhao, Lei Liu

**Affiliations:** ^1^ Nantong Hospital of Traditional Chinese Medicine, Affiliated Traditional Chinese Medicine Hospital of Nantong University, Nantong, China; ^2^ Department of Occupational Medicine and Environmental Toxicology, Nantong Key Laboratory of Environmental Toxicology, School of Public Health, Nantong University, Nantong, China; ^3^ Department of Pathology, Affiliated Hospital of Nantong University, Nantong, China

**Keywords:** glioblastoma multiforme, PXDN, TCGA, immunomodulator, prognosis, immune infiltration

## Abstract

Peroxidase (PXDN), a specific extracellular matrix (ECM)-associated protein, has been determined as a tumor indicator and therapeutic target in various tumors. However, the effects of PXDN in prognostic performance and clinical implications in glioblastoma multiforme (GBM) remains unknown. Here, we assessed PXDN expression pattern and its performance on prognosis among GBM cases from TCGA and CGGA databases. PXDN was up-regulated within GBM samples in comparison with normal control. High PXDN expression was a dismal prognostic indicator in GBM. Single cell RNA analysis was conducted to detect the cell localization of PXDN. We also set up a PPI network to explore the interacting protein associated with PXDN, including TSKU, COL4A1 and COL5A1. Consistently, functional enrichment analysis revealed that several cancer hallmarks were enriched in the GBM cases with high PXDN expression, such as epithelial-mesenchymal transition (EMT), fatty acid metabolism, glycolysis, hypoxia, inflammatory response, and Wnt/beta-catenin signaling pathway. Next, this study analyzed the association of PXDN expression and immunocyte infiltration. PXDN expression was in direct proportion to the infiltrating degrees of NK cells resting, T cells regulatory, M0 macrophage, monocytes and eosinophils. The roles of PXDN on immunity were further estimated by PXDN-associated immunomodulators. In addition, four prognosis-related lncRNAs co-expressed with PXDN were identified. Finally, we observed that PXDN depletion inhibits GBM cell proliferation and migration by *in vitro* experiments. Our data suggested that PXDN has the potential to be a powerful prognostic biomarker, which might offer a basis for developing therapeutic targets for GBM.

## Introduction

Glioblastoma multiforme (GBM) accounts for a frequently occurring primary cancer in the nervous system of adulthood with the highest malignant grade. As classified by the World Health Organization (WHO) classification, GBM has been considered as a Grade IV glioma (Ostrom et al., 2013; Louis et al., 2021). Although multi-mode therapy is greatly successful in the treatment of GBM, including neurosurgery, radiochemotherapy and immunotherapy, GBM has dismal prognostic outcome, with a median survival as short as 15 months (Alifieris and Trafalis, 2015). Several molecular biomarkers have been identified in GBM through genomic analyses. For instance, 1p/19q deletion is a prognostic signature of GBM indicating a superior prognosis. Methyl guanine methyl transferase (MGMT) is another therapeutic effect marker which forecast the sensitivity of temozolomide therapy (Westphal and Lamszus, 2015). In addition, upregulation of epidermal growth factor receptor (EGFR) was observed in more than 30% cases with glioblastoma and suppression of EGFR greatly blocks cancer cells development (Talasila et al., 2013). However, these typical biomarkers could not predict the survival outcome as they are merely used specific parts of GBM patients. Therefore, exploring the GBM mechanism at molecular level and exploiting novel prognostic biomarker is of great necessity.

The application of new immunotherapeutic approaches in GBM treatment is one of the current research hotspots. With advanced research on CNS, it has been shown that CNS tumors can also be infiltrated by lymphocytes of peripheral origin. Moreover, Peripheral immunity may produce a therapeutically meaningful attack on pre-existing GBM (Lim et al., 2018). Recent advances in immunotherapy for glioma have focused on immune checkpoint inhibitors, CAR-T therapy and tumor Vaccine (Wang et al., 2020a). In-depth understanding and elaboration of immunotherapy in the treatment of glioma could facilitate the development of scientific strategies for immunotherapy of GBM in future clinical and basic research.

Peroxidase (PXDN), initially discovered from Drosophila melanogaster in 1994 by Nelson et al. (1994), is a specific protein related to extracellular matrix (ECM). It is a heme-containing peroxidase family member found in basement membranes, and one of its main functions is to catalyze the formation of thionine bonds between hydroxylysine nitrogen and methionine sulfur with the use of hypohalous acids (Bhave et al., 2012; McCall et al., 2014; Dougan et al., 2019). Typically, such an intermolecular bond plays an important role in maintaining basement membrane integrity (Bathish et al., 2020). PXDN also has an essential role in accelerting various cancer types, such as oral squamous cell carcinoma (OSCC), melanoma, prostate cancer (PCa) and ovarian cancer (OC) (Zheng and Liang, 2018; Dougan et al., 2019; Kurihara-Shimomura et al., 2020; Paumann-Page et al., 2021). Nonetheless, its pathogenic function within GBM is still unknown.

The present work focused on investigating the prognostic performance of PXDN in GBM by the public databases. The underlying biological function and possible pathway by which PXDN gets involved in GBM were analyzed by GSEA. Next, CIBERSORT and TISIDB were employed to detect the immune implications of PXDN in GBM. Finally, this study conducted *in vitro* experiments to illustrate the carcinogenic function of PXDN.

## Methods

### Data processing

TCGA-GBM includes RNA-seq data collected from 169 GBM cases together with five healthy controls. GSE108474 (https://www.ncbi.nlm.nih.gov/geo/) were utilized to validate differential PXDN mRNA expression between GBM (*n* = 221) and matched non-carcinoma (*n* = 28) samples. This study also obtained the pathological and clinical information for GBM cases from the TCGA-GBM set (https://portal.gdc.cancer.gov/) and the CGGA dataset (http://www.cgga.org.cn/).

### Assessment of the Prognostic Significance of PXDN in GBM

For illustrating PXDN’s effect on predicting GBM prognosis, we classified cases as 2 groups according to median GBM level. In addition, we adopted the Kaplan-Meier (K-M) method for assessing 5-years overall survival in TCGA and CGGA cohorts. Additionally, we also drew the receiver operating characteristic (ROC) curves for determining PXDN’s predicting ability.

### Pearson correlation analysis of PXDN

Possible PXDN co-expressed lncRNAs and genes were obtained by Pearson correlation analysis using the thresholds of *p* < 0.001 and correlation coefficient |cor| > 0.3.

### Functional annotation for Co-expressed genes of PXDN

PXDN related co-expressed genes were used to investigate the underlying molecular mechanism of PXDN involvement in GBM by conducting Gene Ontology (GO) as well as Kyoto Encyclopedia of Genes and Genomes (KEGG) analyses (Yu et al., 2012). Additionally, this study also built a PXDN-based protein-protein interaction (PPI) network through STRING (https://string-db.org/).

### Immune microenvironment analysis

CIBERSORT is a computational algorithm which can estimate the immune activity of 22 tumor infiltration cell (TIC) types (Newman et al., 2015). To mirror the immune microenvironment of PXDN in GBM, we applied CIBERSORT to calculate fraction of 22 TICs in all cases with GBM. *p* < 0.05 were selected for the following analysis.

### Gene set enrichment analysis

We applied GSEA to examine the underlying biological function related PXDN (Subramanian et al., 2005). We acquired the Hallmark gene sets based on Molecular Signatures Database upon the threshold of normalized *p* < 0.05.

### Single cell analysis

The single cell analysis according to GSE138794 was carried by scTIME Portal (http://sctime.sklehabc.com/unicellular/home), which consists of 10 GBM case’s cells. All samples were imported into Seurat V3 and visualized in UMAP after a standardized quality control process.

### Cell culture and cell transfection

The human healthy NHA astrocytes and U87, A172 GBM cells were provided by the Chinese Institute of Biochemistry and Cell Biology. These cell lines were routinely cultivated within DMEM (KeyGEN BioTECH, China) that contained 10% fetal bovine serum (FBS, GIBCO, USA) under 37°C and 5% CO_2_ conditions. si‐PXDN and corresponding negative control (si‐NC) were obtained from Ribobio (Guangzhou, China). Supplementary Table S1 presents the sequences of si-PXDN. Lipofectamine 3000 reagent (Invitrogen) was adopted for cell transfection in line with specific protocols.

### Quantitative real-time polymerase chain reaction

Trizol reagent (Invitrogen) was utilized for extracting total cellular RNA, whereas the NanoDrop spectrophotometer for adopted for quantification. The cDNA was prepared by adopting Prime Script RT Master Mix reagent (Takara Bio, Dalian, China) in line with specific protocols. Thereafter, this study employed StepOnePlus real-time PCR system (Thermo Fisher Scientific) for amplifying target genes. Supplementary Table S1 displays primer sequences of all genes. The 2^−ΔΔ^Ct method was adopted for calculating relative gene expression, with GAPDH being the endogenous control.

### Cell Counting Kit-8 assay

CCK-8 kit was utilized to evaluate cell proliferation. 96-well plates were inoculated with cells (2 × 10^3^) per well. 4 time points (24, 48, 72, and 96h), all wells were added with CCK-8 solution (10 μl) to incubate for a 2-h period under 37°C, the spectrophotometer was later utilized to measure absorbance (OD) value at 450 nm. Each assay was carried out in triplicate.

### Colony formation assay

In this assay, we inoculated cells (1 × 10^3^) in 6-well plates to incubate for a 2-week period under 37°C. Thereafter, colonies were subject to 4% paraformaldehyde (500 μl) fixation for a 20-min period as well as 0.1% crystal violet (Beyotime Biotechnology) staining for another 20-min period. Finally, we count the colony number and took photos.

### Cell migration assay

Transwell chambers (24-well plates with 8.0 μm pores; Corning) were employed for transwell assays. In brief, we incubated the 24-well plates under 37°C and 5% CO2 conditions for a 24 h period. After discarding upper cells, the chambers were washed in PBS, followed by 30 min of 4%% methanol fixation and another 30 min of crystal violet staining. Then, five fields were randomly analyzed under the microscope to observe and count the number of infiltrating cells. The experiments were conducted in triplicate.

### Statistical analysis

Kaplan-Meier analysis and receiver operating characteristic (ROC) analysis were performed to examine the reliability of the model. All statistical data were analyzed using GraphPad 8.0 and the R software version 4.0.

## Results

### Expression pattern and prognostic power of PXDN in GBM

**FIGURE 1:**
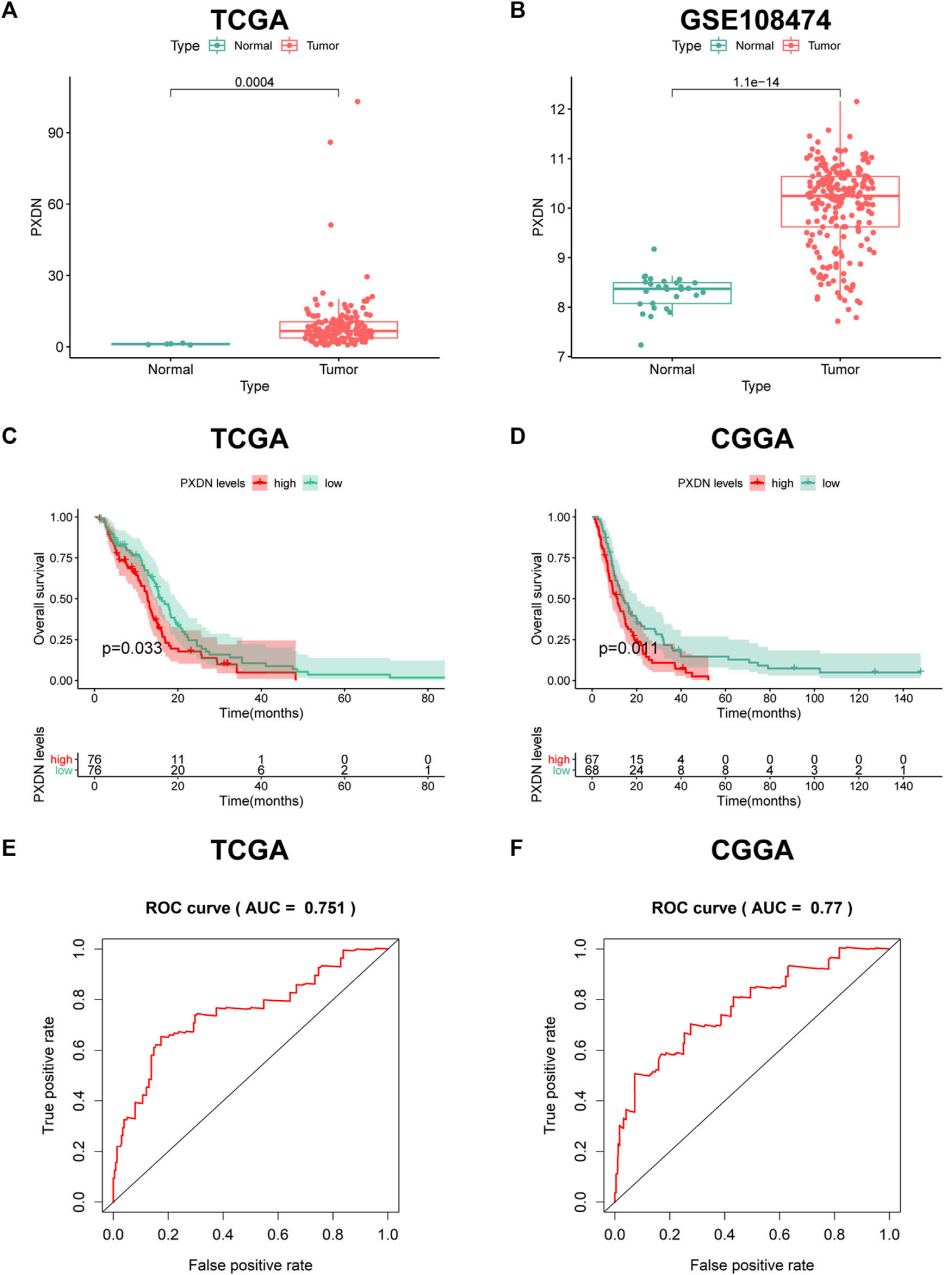
Expression pattern and prognostic value of PXDN in GBM. **(A,B)** Differential PXDN expression in GBM tissues and normal samples. **(C,D)** Survival analysis for PXDN based on KM curves. **(E,F)** ROC curves for assessing the predictive ability of PXDN.

To explore the expression pattern of PXDN in GBM samples and normal tissues, we conducted limma package to analyze gene expression profiling from TCGA-GBM and GSE108474. PXDN expression significantly increased within GBM samples compared with normal controls (Figure 1A). The similar result was also verified in GSE108474 (Figure 1B). Next, we further estimate the prognostic value of PXDN in GBM based on OS information of cases in TCGA and CGGA. The results showed that GBM cases showing PXDN up-regulation had remarkably poor OS compared with those with PXDN down-regulation (Figures 1C,D). Moreover, this study also drew ROC curves for identifying the prognostic value of PXDN expression by analyzing values of area under the curve (AUC) with regard to 5-year survival rate. As shown in Figures 1E,F, the AUC values for CGGA and TCGA datasets were determined to be 0.770 and 0.751, separately.

### Cell localization of PXDN

**FIGURE 2:**
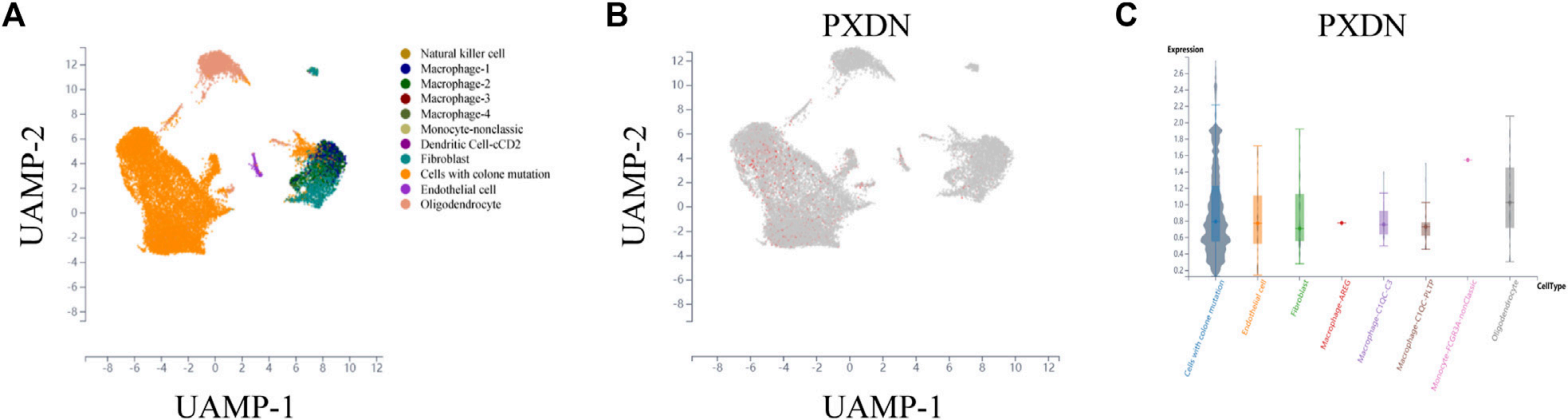
Single-cell sequencing analysis to detect the cell localization of PXDN. **(A)** All cells in GBM samples were clustered into 11 clusters. **(B)** PXDN was mainly enriched in the cell cluster with colone mutation. **(C)** Violin diagram showing the PXDN expression in different cell clusters.

To further detect the expression pattern of PXDN in the GBM microenvironment, the scTIME portal was applied. We first clustered all the cells into 11 clusters by KNN clustering algorithm (Figure 2A). As shown in Figure 2B, we observed that PXDN was mainly enriched in the cell cluster with colone mutation. In addition, violin diagram suggested that PXDN was most highly expressed in monocyte-nonclassic cells (Figure 2C).

### Construction of PXDN associated PPI network

**FIGURE 3:**
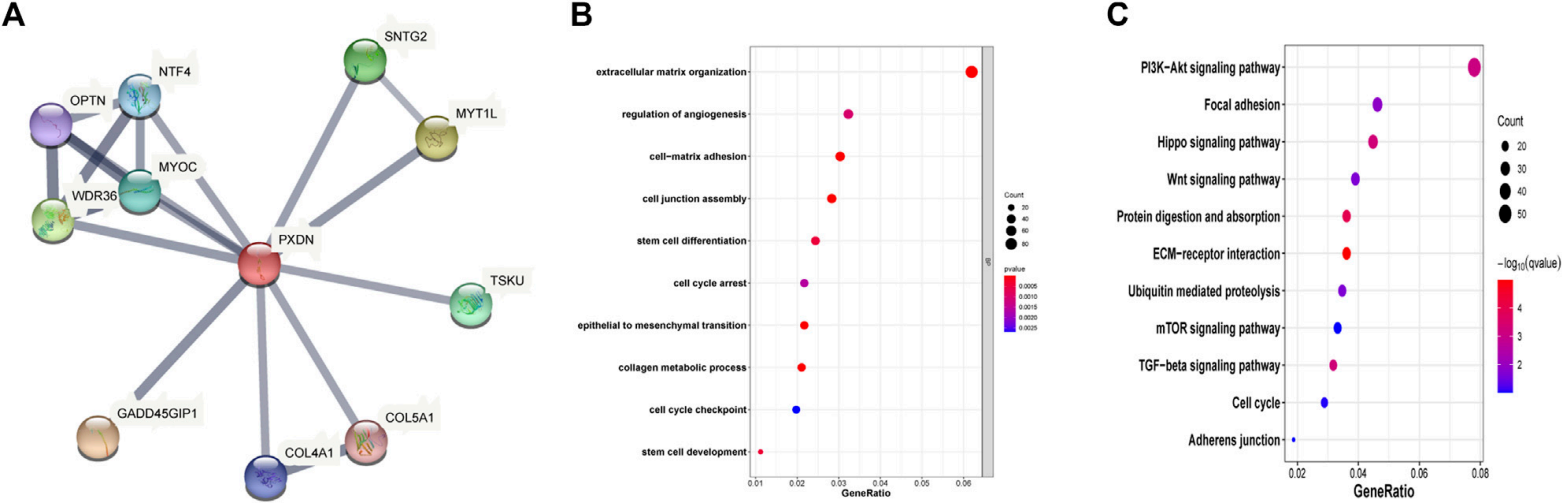
Function analysis of PXDN. **(A)** Construction of PXDN associated PPI network. **(B)** GO biological analysis **(C)** KEGG pathway enrichment.

This study also built the PXDN-associated PPI network based on STRING for examining those interactive proteins, which involved 18 edges, 11 nodes, with the mean coefficient of local clustering being 0.925. The potential interacting genes including NTF4, OPTN, WDR36, MYOC, SNTG2, MYT1L, TSKU, GADD45GIP1, COL4A1 and COL5A1 (Figure 3A). GO analysis showed that PXDN was greatly associated with the regulation of angiogenesis, cell junction and cell cycle arrest (Figure 3B). Moreover, PXDN was bound up with several classic cancer pathways including PI3K/Akt pathway, Hippo signaling and Wnt pathway (Figure 3C).

### PXDN related gene set enrichment

**FIGURE 4:**
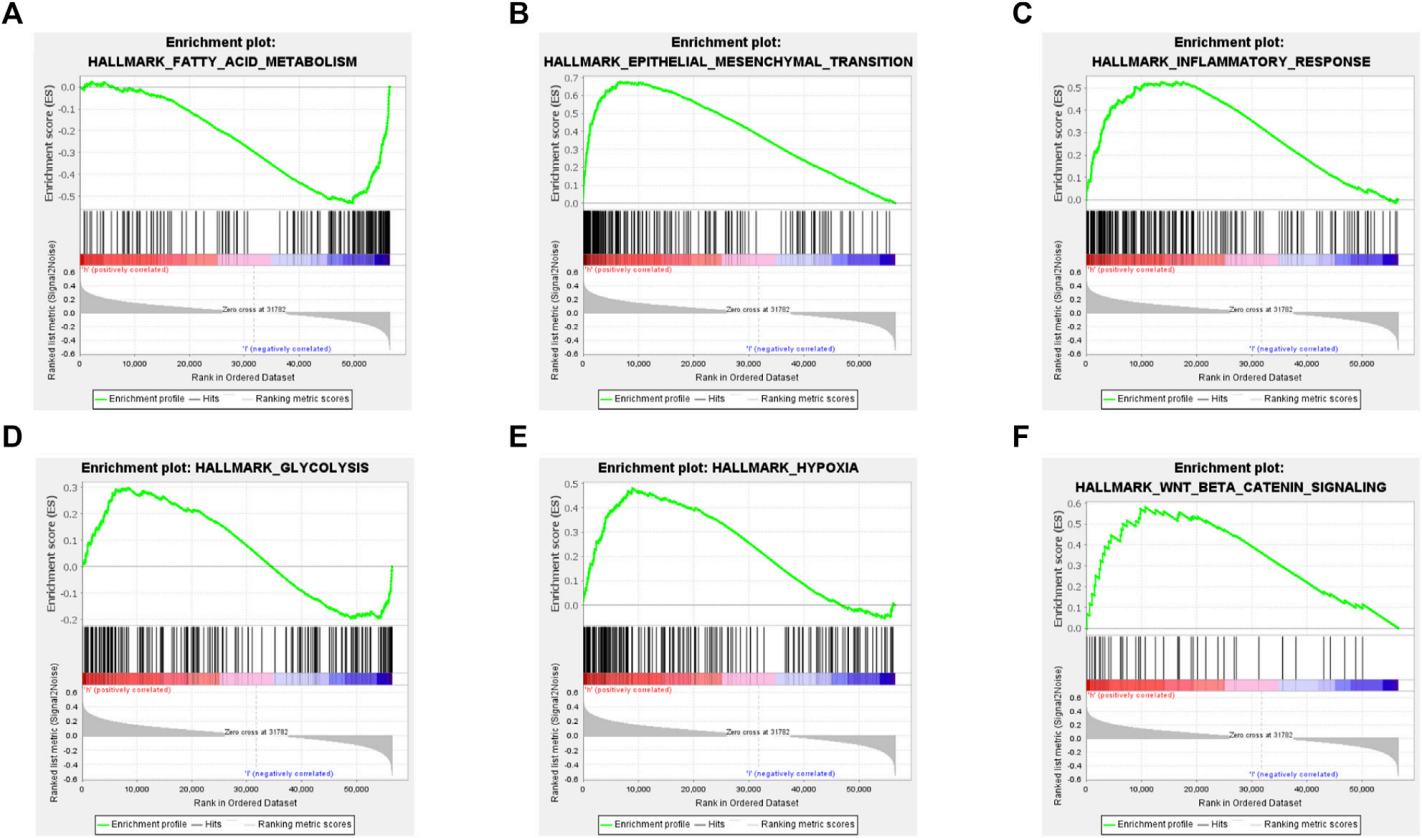
Gene set enrichment analysis of PXDN. **(A)** Fatty acid metabolism. **(B)** Epithelial-mesenchymal transition. **(C)** Inflammatory response. **(D)** Glycolysis. **(E)** Hypoxia. **(F)** Wnt/beta-catenin pathway.

By performing GSEA, we determined hallmark gene set enriched in PXDN high expression group. The results revealed the significant activation of epithelial-mesenchymal transition (EMT), fatty acid metabolism, inflammatory response, Wnt/beta-catenin pathway, hypoxia and glycolysis in PXDN high expression group (Figure 4).

### Association between PXDN and TICs and immunomodulators

**FIGURE 5:**
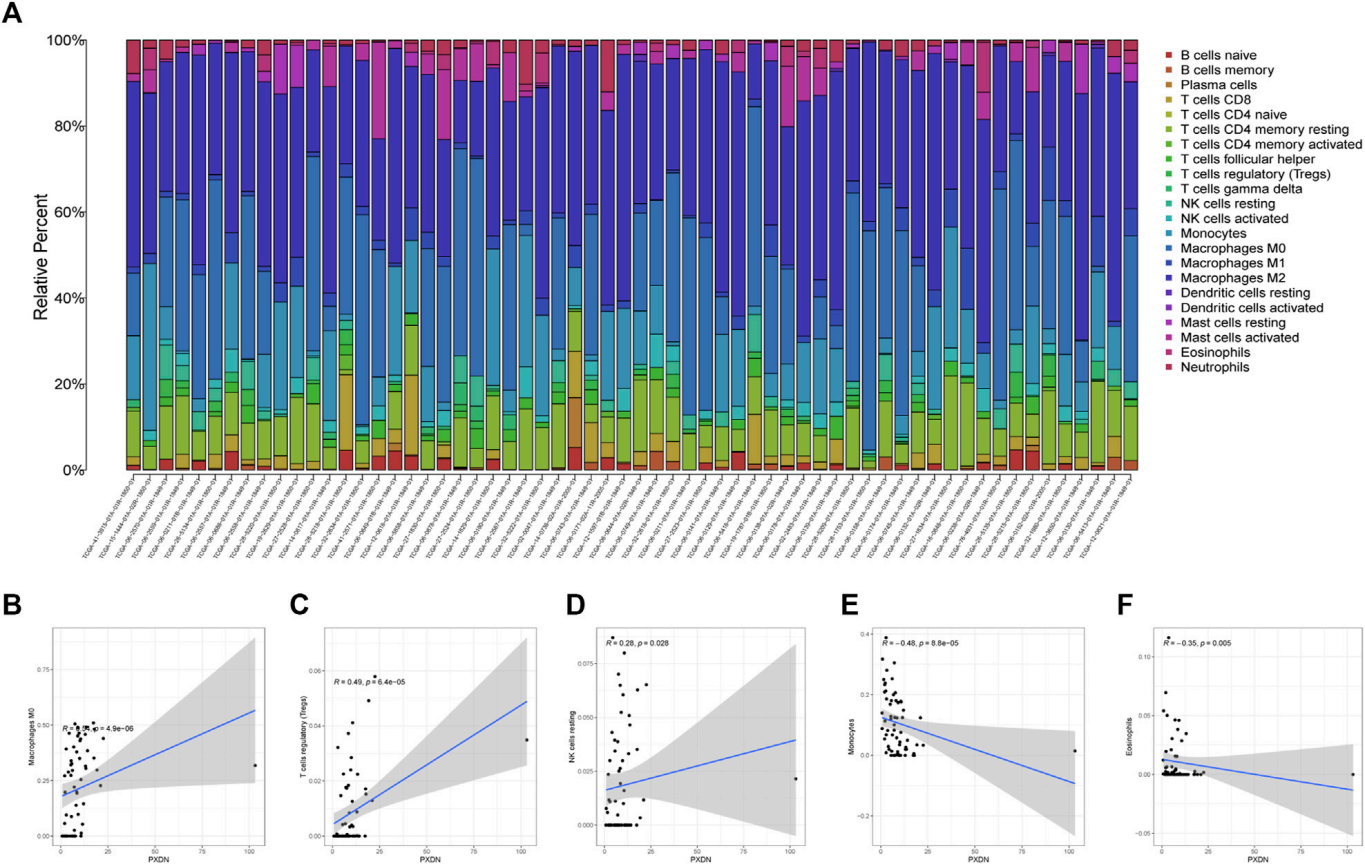
Immunocyte infiltration analysis. **(A)** Immune cells landscape of all GBM samples. **(B–F)** Correlation analysis of immunocyte and PXDN expression (M0 macrophage, T cells regulatory, NK cells resting, monocytes and eosinophils).

**FIGURE 6:**
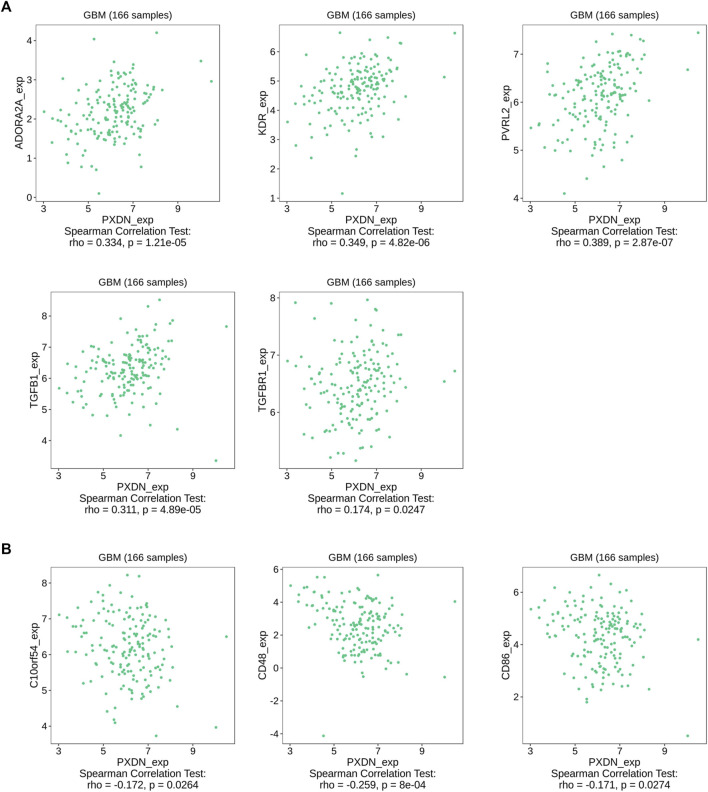
Association between PXDN and immunomodulators. **(A)** PXDN-related immunoinhibitors. **(B)** PXDN-related immunostimulators.

The proportions of 22 immune cell types within GBM cases obtained based on the CIBERSORT algorithm and the results of all tumor samples were shown using a barplot (Figure 5A). As a result, PXDN level showed positive correlation with M0 macrophage (R = 0.54), T cells regulatory (Tregs, R = 0.49) and NK cells resting (R = 0.28), whereas negative correlation with monocytes (R = 0.48) and eosinophils (R = 0.35, Figures 5B–F). According to the TISIDB tool, we identified five immunoinhibitors (ADORA2A, KDR, PVRL2, TGFB1 and TGFBR1) and three immunostimulators (C10orf54, CD48 and CD86) that were significantly associated with PXDN expression in GBM (Figure 6).

### Association of PXDN expression with m^6^A-Related markers

**FIGURE 7:**
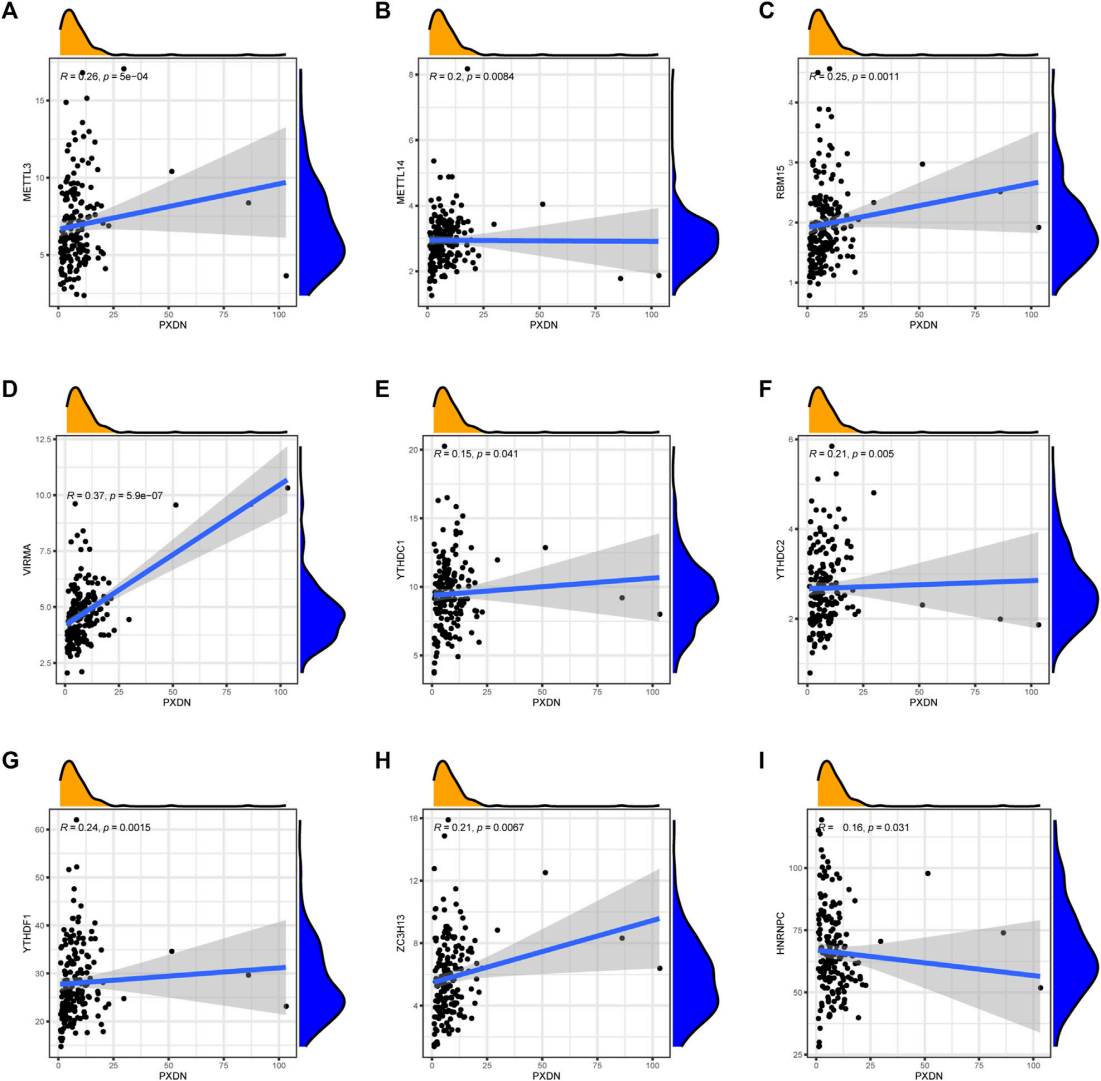
Association of PXDN expression with m6A-related markers. **(A)** METTL3. **(B)** METTL14. **(C)** RBM15. **(D)** VIRMA. **(E)** YTHDC1. **(F)** YTHDC2. **(G)** YTHDF1. **(H)** ZC3H13. **(I)** HNRNPC.

To explore the relationship between PXDN and m6A-related markers, we conducted Spearman correlation analysis. We found that the expression of PXDN was METTL3 (R = 0.26), METTL14 (R = 0.20), RBM15 (R = 0.25), VIRMA (R = 0.37), YTHDC1 (R = 0.15), YTHDC2 (R = 0.21), YTHDF1 (R = 0.24) and ZC3H13 (R = 0.21). Nevertheless, only HNRNPC showed a negative correlation with PXDN (R = 0.16, Figure 7).

### Analysis of lncRNA Co-expressed with PXDN

**FIGURE 8:**
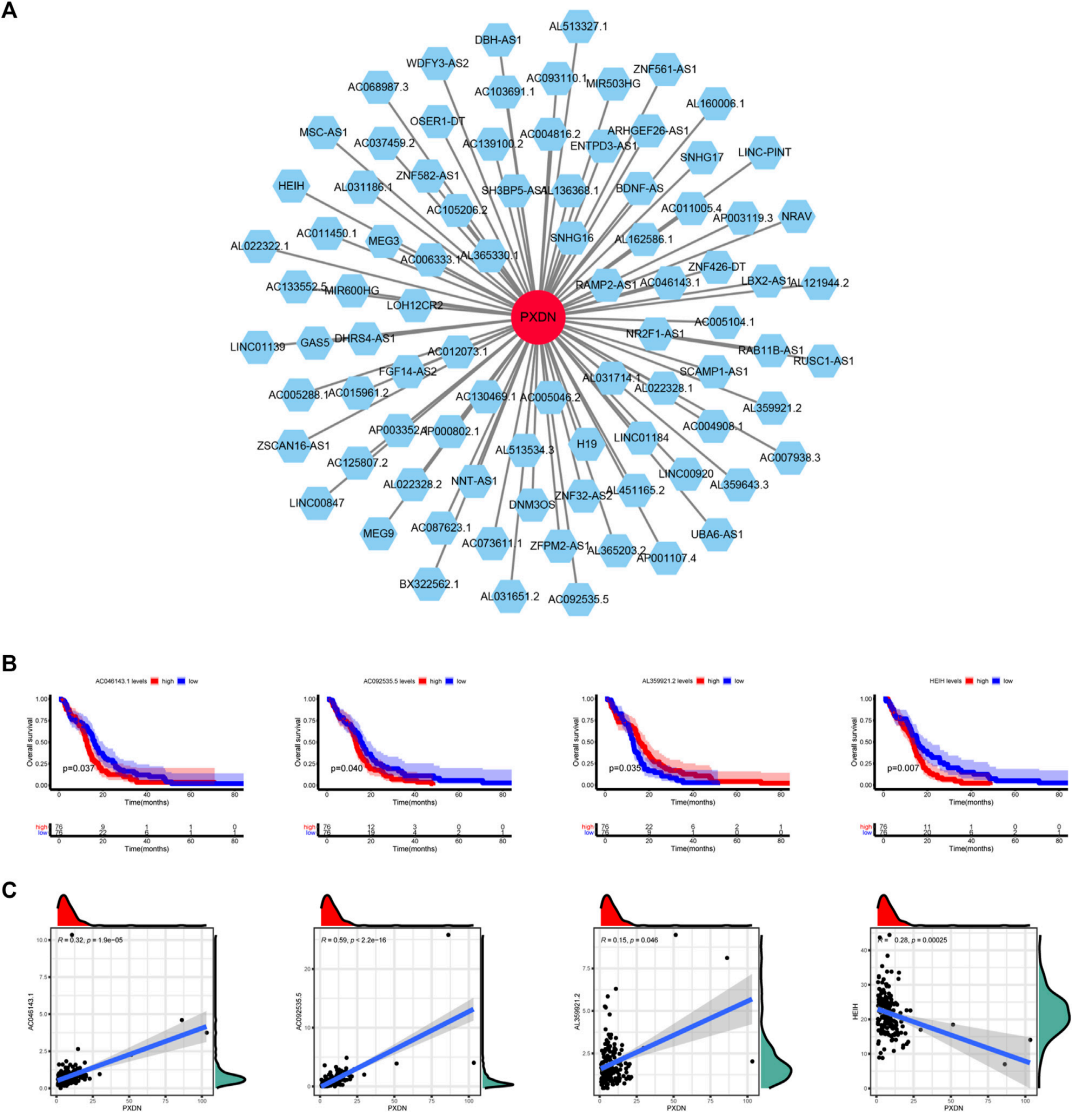
Determination of PXDN-related lncRNAs. **(A)** PXND-lncRNAs correlation regulation network. **(B)** Survival analysis of four lncRNAs. **(C)** Correlation analysis of four lncRNAs.

The potential lncRNAs co-expressed with PXDN were identified by Spearman correlation analysis (Figure 8A). We further used K-M survival method to determine the potential prognostic performance of lncRNAs. As shown in Figure 8B, GBM cases who had AL359921.2 up-regulation had markedly superior prognosis to patients with AL359921.2 down-regulation. However, AC046143.1, AC092535.5 and HEIH presented positive relationship between high expression and dismal clinical outcome (Figure 8B). Figure 8C illustrated the correlation between PXDN and four prognosis-related lncRNAs.

### Silencing of PXDN blocked GBM cell proliferation and migration

**FIGURE 9:**
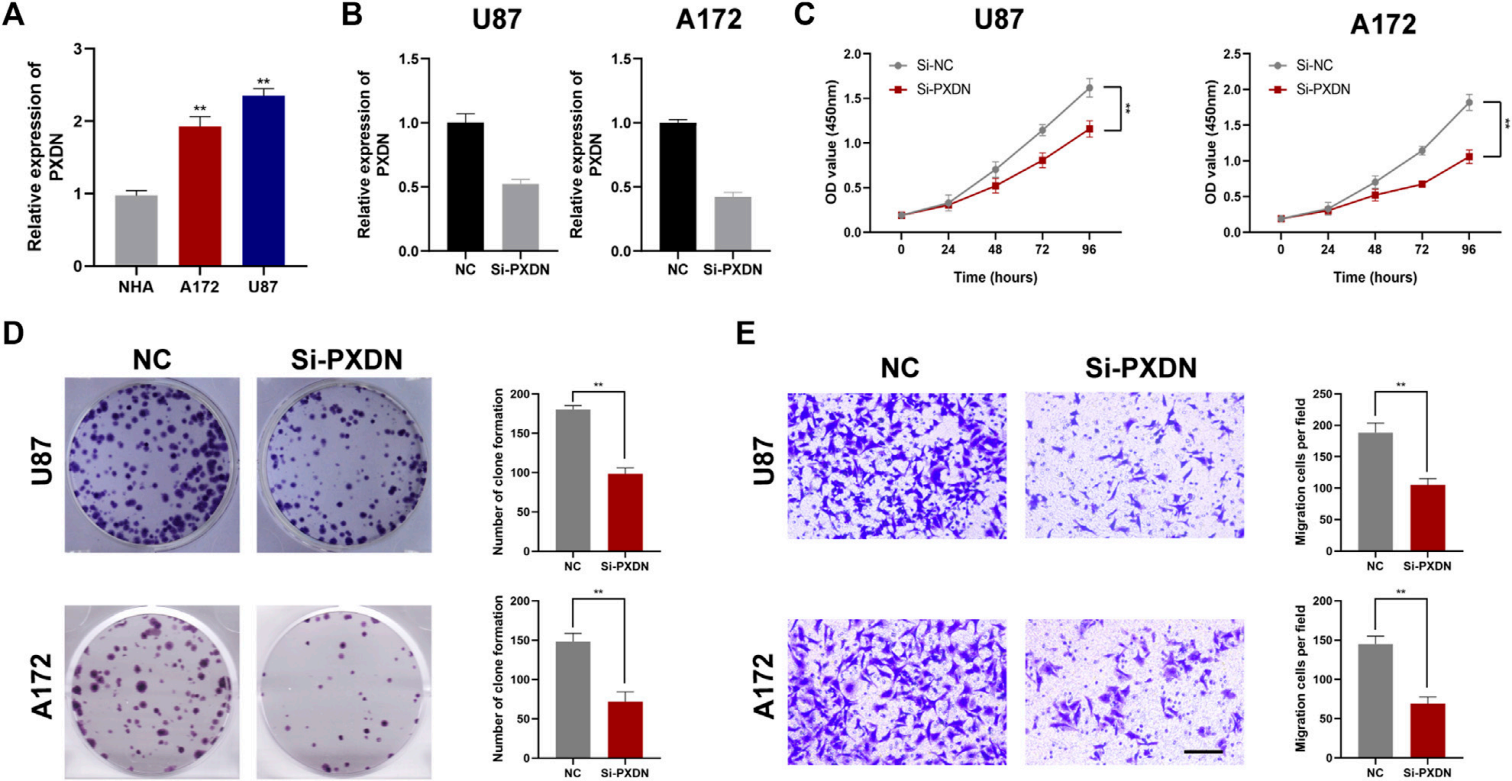
Silencing of PXDN blocked GBM cell proliferation. **(A)** The mRNA expression level of PXDN in NHA and GBM cell lines. **(B)** qRT-PCR to assess the silencing effect of PXDN after siRNA transfection. **(C,D)** The effect of PXDN on proliferation in U87 and A172 was examined using CCK-8 assay and clone formation. **(E)** The role of PXDN on migration in U87 and A172 was detected by tranwell assay.

To be started, we verified expression level of PXDN between GBM (U87, A172) and NHA cells by qRT-PCR. As shown in Figure 9A, PXDN was upregulated in GBM cells relative to NHA cells, especially in the U87 cell line. Next, we applied siRNAs for inhibiting PXDN within GBM cells and performed qRT-PCR analysis to confirm its efficacy (Figure 9B). We found that the inhibition of PXDN expression dramatically suppressed GBM cell proliferation, which was demonstrated in CCK8 proliferation assay and colony formation assay (Figures 9C,D). We also assessed the effect of silencing PXDN on GBM cell migration. Significantly, loss of PXDN decreased the migration of GBM cells (Figure 9E).

## Discussion

GBM is one of the most common and most malignant primary nervous system diseases that threatens global health. Due to the shortage of identified specific biomarkers, patients with GBM are diagnosed at late stage, which lead to dismal clinical outcome. Therefore, the determination of novel biomarkers for GBM has become an urgent priority in clinical practice. In our study, we first observed that PXDN has higher expression level in GBM tissues relative to normal counterparts. Additionally, we obtained potential interacting proteins of PXDN from PPI network by string tool. Moreover, PXDN was found to be associated with immunocyte infiltration in GBM. Furthermore, we observed that silencing PXDN greatly inhibited GBM cell growth and migration by *in vitro* experiments.

Previous studies reported that PXDN was significantly upregulated in oral squamous cell carcinoma and ovarian cancer (Zheng and Liang, 2018; Kurihara-Shimomura et al., 2020). However, the role of PXDN in GBM is unclear. We observed that PXDN expression in GBM samples was distinctly lower than in normal cases. Moreover, PXDN up-regulation within GBM predicted poor prognostic outcome.

In addition, PPI network was constructed and 11 potential interacting genes were identified, including NTF4, OPTN, WDR36, MYOC, SNTG2, MYT1L, TSKU, GADD45, GIP1, COL4A1 and COL5A1. It was reported that a few of these genes are closely bound up to tumorigenesis and cancer therapeutics. For example, NTF4 is upregulated in colorectal cancer and mediates CRC development through regulation of EMT and autophagy (Yang et al., 2020). In neuroblastoma and non-small cell lung cancer (NSCLC), high expression of TSKU was negatively correlated with patients’ prognosis (Zhao et al., 2018; Huang et al., 2021). COL4A1, which belongs to collagen family, accounts for an essential part of ECM structure discovered in many embryonic and connective tissues. As revealed by Wang et al., COL4A1 boosts proliferation, hepatocellular carcinoma (HCC) cell invasion and migration through the activation of FAK-Src pathway, which suggested that COL4A1 was the possible diagnostic and therapeutic marker for HCC (Wang et al., 2020b). Its cancer-promoting role in HCC was also confirmed by Zhang et al. (Zhang et al., 2021a). In addition, COL5A1 up-regulation predicted dismal prognostic outcome in renal clear cell carcinoma (RCCC), BC, OC, and gastric cancer (GC) (Feng et al., 2019; Wei et al., 2020; Zhao et al., 2020; Zhang et al., 2021b).

To exploit the underlying mechanism of PXDN in GBM, we performed GSEA analysis. The results indicated that PXDN mainly regulates GBM development by activating cancer hallmarks, including fatty acid metabolism, epithelial-mesenchymal transition, inflammatory response, glycolysis, hypoxia and Wnt/beta-catenin signaling pathway. Epithelial-mesenchymal transition (EMT) is a reversible cellular process, which keeps cells in a transitional state between partial epithelium and partial mesenchyme. The activation of EMT could lead to the loss of polarity of epithelial cells, the dissolution of intercellular junctions, the acquisition of motor ability and the reorganization of extracellular matrix (ECM) (Dominguez et al., 2017; Shibue and Weinberg, 2017). According to the current research, EMT not only improves the tolerance of tumors to treatment, but also gives cancer cells greater tumorigenicity and metastatic potential (Dongre and Weinberg, 2019). Quite a number of experiments have shown that EMT can be promoted or inhibited through a variety of pathways, and the process of EMT is positively correlated with the degree of GBM invasion (Lu et al., 2015; Polonen et al., 2019; Yang et al., 2019; Pan et al., 2020). Perhaps because oxygen is not available to meet the demands of the rapidly growing tumor, GBM tumor tissue is characterized by widespread hypoxia which could induce the expression of Hypoxia Inducible Factor (HIF) (Mennerich et al., 2019). Interestingly, hypoxia is an important environmental factor for glioma stem cell (GSC) survival, which is also associated with invasion, new vessel formation and radioresistance of tumor (Colwell et al., 2017). Wnt/β-catenin pathway represents the highly conservative signal cascade, which participates in various biological processes, like cell growth, migration, apoptosis, differentiation, and tissue homeostasis. Dysfunctional miR22HG promotes GBM invasiveness and GSC carcinogenesis through Wnt/β-catenin signal pathway (Han et al., 2020). Xiaoping Zhu et al. found that Moesin could promote GBM cell growth and activate Wnt/β-catenin pathway by interacting with CD44 (Zhu et al., 2013). Moreover, RPN2 regulated glioma development and mediated temozolomide sensitivity through Wnt/β-catenin pathway (Sun et al., 2020).

Immunotherapy is an effective novel therapy for a number of tumors. However, GBM has very little benefit on immunotherapy. Tumor resistance to immunotherapy is driven by internal and external factors that lead to immune evasion, including myeloid derived suppressor cells, like tumor-associated macrophages (TAMs) and regulatory T cells (Tregs) (Gomez et al., 2020). Tregs, a subpopulation of CD4^+^ T cells, suppress immunity by secreting cytokines that suppress effector T cells, maintain immune homeostasis and prevent the development of autoimmune diseases (Gomez and Kruse, 2006; Nakagawa et al., 2016). Previous reports have shown that up to 60% of the tumor-infiltrating lymphocyte (TIL) population in tumor tissue is composed of Treg, a proportion substantially higher than the proportion of circulating Treg cells in high-grade glioma (Sakaguchi, 2005). Abundant infiltration of Tregs may contribute to the defective T cell proliferation as well as to GBM progression. In GBM, infiltration and enrichment of TAMs is a common characteristic (Komohara et al., 2008; Shi et al., 2015). Furthermore, TAMs are more likely to polarize to an immunosuppressive M2-like phenotype (Hussain et al., 2006; Fu et al., 2020). High expression of M2-like TAM markers (CD204 and CD163) in GBM predicts dismal prognostic outcome and aggressive phenotype of glioma (Andersen et al., 2021).

To further explore PXDN immune implication in GBM, we identified PXDN-associated immunomodulators by TISIDB database. KDR, a kinase insert domain receptor of the VEGF, could regulate tumor progression and angiogenesis. As suggested by Wu et al., KDR activation could be induced by autophagy, which in turn facilitates tumor vasculogenic formation by glioma stem cells (Wu et al., 2017). In addition, KDR, a target gene for miR-497 in lung cancer, could boost cancer cell growth and inhibit cell apoptosis (Xia et al., 2019). PVRL2, a novel immune checkpoint, may inhibit PD-L1-T cell activity in various tumors, such as endometrial carcinoma, lung cancer, ovarian cancer and breast cancer (Whelan et al., 2019). In our analyses, we found that PXDN was positively correlated with KDR, suggesting that PXDN might promote GBM development by KDR or PVRL2 related pathways.

Accumulating evidence has suggested that the ectopic expression of lncRNAs in various tumor cells could facilitate tumorigenesis, tumor development and metastasis (Bhan et al., 2017; Li et al., 2018; Jiang et al., 2019). Therefore, we further determined four prognosis-related lncRNAs co-expressed with PXDN. Among these potential lncRNAs, AC046143.1 and HEIH have been previously proved to be associated with cancer. In GBM, AC046143.1 was used to set up an immune-related biomarker signature for risk classification and prognosis prediction (Li et al., 2021). Numerous reports have revealed that HEIH play a central part in all kinds of tumors, including hepatocellular carcinoma, cholangiocarcinoma and esophageal cancer (Wang et al., 2020c; Shen et al., 2020; Wan et al., 2020). In cholangiocarcinoma, HEIH was found to enhance cell viability and metastasis via miR-98-5p/HECTD4. As unearthed by Wang et al., HEIH knockdown suppresses malignant behavior in esophageal cancer by targeting miR-185/KLK5 (Wang et al., 2020c).

The N6-methyladenosine (m6A) modification plays a central part in tumorigenesis and cancer progression. Li et al. showed that inhibiting METTL3 could block the proliferation and self-renewal of glioma stem cells (GSC), suggesting upregulated METTL3 leads to highly aggressive GBM (Li et al., 2019). YTHDF1, a methylation recognition protein, could specifically bind m6A-containing mRNAs and modulates their stability. As suggested by Wang et al., Musashi-1 could enhance the GSC properties of GBM by targeting YTHDF1. They observed that the YTHDF1 expression could be positively affected by inhibition or overexpression of Musashi-1 and silencing of YTHDF1 could repressed the growth and chemoresistance of GBM cells (Yarmishyn et al., 2020). Our data indicated that PXDN expression was positively associated with the expressions of METTL3 and YTHDF1. Consequently, we speculate that PXDN might regulate GBM survival and development by METTL3 and YTHDF1 in a m6A modification way.

As far as we know, the present work is the first to investigate prognostic value and clinical implications in GBM based on bioinformatic methods. First, our study was mainly based on online databases. Moreover, the expression pattern of PXDN needs to be verified in the local cohorts. We will further explore the possible mechanism of PXDN by on oncogenic effects.

## Conclusion

In summary, our data revealed that PXDN is upregulated in GBM samples, while high PXDN expression predicts a poor prognosis. PXDN expression is associated with the several immunocyte infiltration, such as M0 macrophage, T cells regulatory, NK cells resting, eosinophils and monocytes. Furthermore, we observed that PXDN depletion inhibits GBM cell proliferation and migration, which might offer a basis for developing therapeutic targets for GBM.

## Data Availability

The original contributions presented in the study are included in the article/Supplementary Material, further inquiries can be directed to the corresponding authors.
