# Supplementary material for: Peroxidase is a novel potential marker in glioblastoma through bioinformatics method and experimental validation
Source: Front Genet. 2022 Aug 31;13:990344. doi: 10.3389/fgene.2022.990344 (PMC9471987; doi:10.3389/fgene.2022.990344)
Supplement: Supplementary file 1 [file Table1.DOCX]

Supplementary Table 1 Primer and siRNA sequences for each gene.

| Gene | Primer sequence | |
| --- | --- | --- |
| GAPDH | Forward | 5′-CTGGGCTACACTGAGCACC-3′ |
|  | Reverse | 5′-AAGTGGTCGTTGAGGGCAATG-3′ |
| PXDN | Forward | 5′-CAGGGACCTCGTTAATGGCT-3′ |
|  | Reverse | 5′-CCGTTCAGACAGCTGACGTT-3' |
| Gene | siRNA sequence | |
| si-NC | 5′-AUGAAAUGGGAUCAAGUGG-3′ | |
| si-PXDN | 5′-AACAGGGCAGAAAGUUGUA-3′ | |
